# Evaluating social–emotional skills among Arabic‐speaking students: A multi‐informant study of students, parents and teachers

**DOI:** 10.1111/bjep.70044

**Published:** 2025-11-11

**Authors:** Ibrahim Asadi

**Affiliations:** ^1^ The Academic Arab College for Education Haifa Israel; ^2^ The Unit for the Study of Arabic Language, Edmond J. Safra Brain Research Center for the Study of Learning Disabilities Haifa Israel

**Keywords:** Arabic‐speaking, discrepancy, self‐report questionnaires, social–emotional learning skills

## Abstract

**Background:**

Social–emotional (SEL) skills play a crucial role in promoting children's academic performance and mental health. However, the use of self‐reported questionnaires may reveal discrepancies between students' self‐assessments and adults.

**Aims:**

This study investigated potential differences in the perceptions of SEL skills in Arabic and their relation to academic achievements.

**Sample and Method:**

Arabic‐speaking students, their parents and teachers across third, fifth, seventh and ninth grades using a 33‐item questionnaire.

**Results:**

Analysis of variance (ANOVA) revealed significant discrepancies between groups (parents>students>teachers), regardless of grade level. Moreover, correlations between self‐reports and students' academic scores were stronger for teachers compared to the others. Interestingly, these correlations decreased across groups until the ninth grade, at which point only teachers maintained significant correlations. Structural equation modelling (SEM) analysis further demonstrated that only teachers' self‐reports significantly predicted students' academic scores regardless of grade level.

**Conclusion:**

The results are discussed in relation to theoretical and pedagogical implications, as well as previous findings, emphasizing the need for a standardized multi‐informant questionnaire.

## INTRODUCTION

Social–emotional learning (SEL) skills are widely recognized as critical to children's academic outcomes and their well‐being and success in life (Durlak et al., 2022; Prince, 2024). However, evaluating SEL skills among students presents unique challenges, especially when using self‐reported questionnaires that may not fully reflect students' thoughts and feelings. Research evaluating parents' and teachers' perceptions of students' SEL skills has shown low to moderate agreement with students' ratings (Caqueo‐Urízar et al., 2022; De Los Reyes et al., 2015; De Los Reyes & Kazdin, 2005). In addition, SEL development is shaped not only by specific school contexts but also by broader sociopolitical and cultural factors (Agbaria, 2024; Osher et al., 2020). Emerging research, therefore, highlights the necessity of adapting SEL models to cultural contexts to enhance the effectiveness of both assessment and intervention (Agbaria, 2024; Li et al., 2025).

To date, no research has tested the possible discrepancy in self‐reported questionnaires between students and adults in Arabic. Specifically, there is a dearth of research on how Arabic‐speaking students' perceptions of SEL skills align with their parents' and teachers' perceptions, especially across elementary and middle schools. Understanding these potential discrepancies is critical, as students' self‐perceptions may impact their behaviour and development (Harter, 2012). Moreover, testing these discrepancies will contribute to the literature on multi‐informant assessments and may inform the development of comprehensive assessment practices and targeted interventions—particularly given the unique cultural and contextual dimensions of SEL within Arab communities in Israel (Agbaria, 2020, 2024). Accordingly, this study aims to test potential discrepancies between students', parents' and teachers' perceptions of SEL skills in elementary and middle schools, as well as examine how these discrepancies may relate to students' academic achievements. Our main research questions are as follows: How do students' perceptions of their SEL skills differ from their teachers' and parents' perceptions? And whether the discrepancies are related to students' achievements?

## THEORETICAL BACKGROUND

Social–emotional learning (SEL) has emerged as a critical component of education and child development in recent decades (Hoskins & Schweig, 2024). SEL represents the process of developing students' capacity to master skills required for managing their emotions, establishing and maintaining positive relationships with others and making responsible decisions—all of which are necessary not only for life in general but also for academic success (Hoskins & Schweig, 2024; Müller et al., 2024). Initially promoted by the Collaborative for Academic, Social and Emotional Learning (CASEL, 2013), SEL encompasses five key skills: self‐awareness, self‐regulation (or self‐management), social awareness, relationship skills and responsible decision‐making. These skills have been found to correlate with mental health, well‐being and academic achievement (Hayashi et al., 2022).

Although the CASEL framework enjoys international recognition, scholars have underscored the importance of culturally adapting SEL models, especially within minority and non‐Western contexts (Hayashi et al., 2022; Li et al., 2025). In Arabic‐speaking communities, SEL development may be influenced by unique socio‐cultural dynamics such as religious identity, minority status and collectivist values within Israel—factors that necessitate culturally tailored assessment instruments (Majadly & Haj Yahya, 2024). Accordingly, this study examines the perceptions of students, parents and teachers regarding SEL within the context of Arab communities in Israel.

The developmental trajectory of social–emotional skills is complex, with various competencies evolving at different rates and influenced by distinct factors throughout childhood and adolescence (Denham et al., 2009). Studies examining different developmental aspects of social–emotional skills have yielded inconsistent findings. Some have shown a clear developmental effect, with higher scores on emotional and social skills associated with increasing age (Bredikyte & Brandisauskiene, 2023; Edossa et al., 2018). In contrast, others have reported that younger students score higher than older ones (Burns & Rapee, 2016; Feraco & Meneghetti, 2023; West et al., 2020).

A substantial body of research has demonstrated a positive relationship between SEL and academic performance, as well as overall well‐being (Hoskins & Schweig, 2024; Müller et al., 2024). This relationship is further supported by intervention studies showing that students who participate in social–emotional intervention programmes also experience enhancements in their academic performance (Durlak et al., 2011; Taylor et al., 2017). These findings suggest that social and emotional skills contribute to students' sense of security and safety, improve their feelings of connection and belonging to school, and foster better relationships with peers and teachers (Bergin & Bergin, 2009; Blair & Raver, 2015). Consequently, these factors may enhance students' motivation and engagement in learning activities.

The positive impact of SEL on the learning process has been documented across various age groups. In early childhood (Müller et al., 2024), a meta‐analysis of 48 studies on preschoolers (Murano et al., 2020) and a systematic review of 79 studies on students aged 5–8 years (Blewitt et al., 2018) demonstrated that young students involved in SEL programmes showed improvements in various aspects of social–emotional development as well as early learning outcomes. In recent years, several school‐based SEL programmes have been implemented and evaluated in Israel. For instance, the ‘I Can Succeed’ programme, which focuses on strengthening students' communication and interpersonal skills to enhance social–emotional competencies, was tested among 419 fourth‐grade students over two academic years. The results revealed significant improvements in both interpersonal skills and emotional functioning (Kopelman‐Rubin et al., 2021). The relationship between social–emotional skills and learning performance has also been observed among students in middle and high schools (Ayllón‐Salas et al., 2024; Carnazzo et al., 2019) and among university students (Zawadka et al., 2021), suggesting a cumulative effect on both academic and SEL skills.

Evaluating SEL interventions is crucial for understanding their efficacy. One of the most prevalent methods used in this domain, both for assessing intervention programmes and in school settings, is the self‐reported questionnaire. This method offers several advantages, including low cost, ease of administration, and, most importantly, the ability to capture individuals' subjective experiences (Duckworth & Yeager, 2015). Self‐reported questionnaires typically consist of Likert‐scale items that ask participants to rate their agreement with statements related to their feelings, thoughts and behaviours associated with SEL skills. However, it is important to note that despite the critical role of self‐reported questionnaires in assessing students' SEL skills, these measures may not always accurately reflect the objectively measured ability. This limitation underscores the need for a more comprehensive approach to SEL evaluation that incorporates multiple informant sources.

In this context, several studies have reported discrepancies between students' self‐reporting of SEL skills and ratings provided by parents and teachers (Caqueo‐Urízar et al., 2022; De Los Reyes et al., 2015). A meta‐analysis by Renk and Phares (2004) found only low to moderate correlations between self‐reports of students aged 3–19 years and adult ratings of SEL skills. Similarly weak correlations were observed between students and their teachers (Measelle et al., 2005). Specifically, in elementary school, the correlations between students' self‐reporting of social and behavioural skills and their teachers' ratings were weak, ranging from .22 to .38. A recent study examining discrepancies in social–emotional aspects between 408 elementary school students (aged 8–13 years) and their parents revealed that students reported more difficulties than their parents in various social–emotional aspects, including emotional problems, social anxiety and defiant behaviours (Caqueo‐Urízar et al., 2022).

Such discrepancies may arise from informants' distinct vantage points; students, parents and teachers observe or interpret behaviours within divergent contexts, shaped by their own beliefs, environments and expectations (Brackett et al., 2011; Mudarra et al., 2022). For example, behaviours appropriate in the classroom may differ from those valued at home, and parental expectations may diverge from those held by teachers (Hauser‐Cram et al., 2003). It is worth noting that these discrepancies may also vary based on the cultural characteristics of the population studied.

The Arabic‐speaking community in Israel, comprising 20% of the total Israeli population, is a minority group characterized by a lower socio‐economic status compared to the Jewish community. A clear disparity is evident in the government budget allocation per student between the two education systems (Saffuri, 2025). This disparity is reflected in the number of students per class, the number of teaching hours per week and fewer resources in the educational system for developing non‐cognitive skills, such as SEL skills. Consequently, Arabic‐speaking students face greater challenges in academic performance (Asadi et al., 2017, 2022), as evidenced by their lower scores on international literacy tests compared to Hebrew‐speaking students (OECD, 2019). Moreover, Arab students are disproportionately involved in violent behaviours (Khoury‐Kassabri et al., 2024), despite recent efforts to implement SEL interventions (Benbenishti & Friedman, 2020). It is important to note that SEL development in this community is inextricably linked with socio‐cultural factors—including strong collectivist orientation, complex family structures, religious identity and minority status—highlighting the partial applicability of global, Western‐based SEL models. Cross‐cultural frameworks thus recommend that SEL assessment and intervention be contextually and culturally attuned, acknowledging the influence of specific cultural identities on self‐concept and behaviour (Agbaria, 2020; Ḥaj Yaḥya & Abu‐Baker, 2024; Im, 2023).

Given the paucity of studies concerning SEL skills and their development in Arabic‐speaking populations, coupled with the critical role of SEL skills in academic performance and student development, there is a pressing need for research that examines students' perceptions of their own SEL skills in comparison to their parents' and teachers' perceptions. Such research may illuminate potential discrepancies between students' self‐reporting of SEL skills and adult ratings. Unlike previous studies that compared different respondent sources across various studies, our study is unique in its use of the same method and questionnaires to assess students', parents' and teachers' SEL perceptions within the same sample. This approach may contribute to the development of more accurate and comprehensive assessment methods for SEL skills, which are crucial for developing adequate intervention programmes.

Accordingly, our research questions are as follows:

1. How do students' perceptions of their SEL (composite) skills differ from their teachers' and parents' perceptions across different grade levels?
2. How might the discrepancies between groups in their SEL perceptions relate to students' academic performance?

## MATERIALS AND METHODS

### Participants

The sample comprised 241 students (110 girls and 131 boys), their parents, and their teachers. The students were distributed across various grade levels: 69 third graders, 62 fifth graders, 54 seventh graders and 56 ninth graders. Participants were recruited from four schools (two elementary and two middle schools) representing medium socioeconomic backgrounds in two different villages in northern Israel. In each village, two classes were randomly selected from elementary school (third and fifth grades) and two from middle school (seventh and ninth grades). All students in the selected classes participated in this study, except for those with physical and mental disabilities.

In addition to the students, eight classroom teachers (three males and five females) of the participating students and the respective parents were included in the study. Both teachers and parents were asked to complete the same questionnaire about each student. Of the 241 questionnaires distributed, 236 (97% response rate) were received from teachers and 170 (70% response rate) from parents. All participants were informed about research ethics, emphasizing voluntary participation and the right to withdraw from the study at any time. Students were required to provide a parental consent form to participate in the study. Additionally, the research was approved (AACE‐SE3‐02062025) by the ethical committee of the Arab Academic College of Education in Israel.

### Measures

Social and emotional learning (SEL) development was evaluated using a self‐report online questionnaire (Google Form), which was derived from a previous study (Rezka, 2022) that tested the CASEL model. In a validation study of 336 Arabic‐speaking students, the questionnaire demonstrated high indices of reliability (Cronbach's α ranged from .60 to .81 across different subscales), clarity and validity. The clarity and validity of the items and their adaptation to the different subscales were tested and approved by 12 experts in the field. The content validity ratio (Lawshe, 1975) was used to estimate content validity, with only items achieving a value of at least .56 included in the final questionnaire. Additionally, this questionnaire was tested by confirmatory factor analysis (CFA) to provide empirical and numerical support for its development and quality and to test how well this measure fits a set of empirical data. Findings revealed that all goodness‐of‐fit indices in the measurement model for SEL subscales met the standard values. The model fit indices were as follows: *χ* ^2^_(401)_ = 679.87, *p* < .001, RMSEA = .054, CFI = .90, TLI = .890, SRMR = .060.

The resulting questionnaire consisted of 33 items designed to measure five subscales of the CASEL model:

- Self‐awareness (6 items, *α* = .73, .84, .93 for students, parents and teachers, respectively). Sample item for students: ‘*I feel optimistic about my future*’. Corresponding items for parents and teachers: ‘*My son/daughter feels optimistic about his/her future*’ and ‘*My student feels optimistic about his/her future*’, respectively.
- Self‐regulation or self‐management (6 items, *α* = .75, .76, .92 for students, parents, teachers, respectively). Sample item for students: ‘*I complete my work on time and do not leave it until the last minute’*. Corresponding items for parents and teachers: ‘*My son/daughter completes his/her work on time and does not leave it until the last minute’* and ‘*My student completes his/her work on time and does not leave it until the last minute’*, respectively.
- Social awareness (6 items, *α* = .78, .85, .95 for students, parents, teachers, respectively). Sample item for students: ‘*I listen carefully to others' points of view’*. Corresponding items for parents and teachers: ‘*My son/daughter listens carefully to others' points of view’* and ‘*My student listens carefully to others' points of view’*, respectively.
- Relationship skills (7 items, *α* = .69, .76, .86 for students, parents, teachers, respectively). Sample item for students: ‘*My classmates avoid talking to me’*. Corresponding item for parents and teachers: ‘*His/her classmates avoid talking to him/her’*.
- Responsible decision making (7 items, *α* = .77, .80, .92 for students, parents, teachers, respectively). Sample item for students: ‘*I evaluate the consequences of my choices after each situation’*. Corresponding items for parents and teachers: ‘*My son/daughter evaluates the consequences of his/her choices after each situation’* and ‘*My student evaluates the consequences of his/her choices after each situation’*, respectively.

Mean scores on a 5‐point Likert scale were used to evaluate each item, with affirmative statements followed by five response options for all three groups: 1 = totally disagree, 2 = disagree, 3 = sometimes, 4 = agree, 5 = totally agree. All negatively worded items were reverse‐coded, and thus higher scores indicated higher social–emotional development. The overall reliability of this questionnaire (Cronbach's α) in the current study was .90, .87, .95 for students, teachers, parents, respectively.

In addition, this study examined the correlations between students', parents', teachers' SEL reporting and students' academic scores in three core academic domains: Arabic language, mathematics, science, as reported in the school administrative records for the last trimester.

### Procedure

Students completed the online questionnaire in the presence of an examiner in a computer room at the sampled schools. The questionnaire, administered via Google Forms, took students approximately 10–15 minutes to complete. Students with reading difficulties, or those who requested assistance in reading the questionnaire, received support from the examiners. All examiners were master's degree students in linguistics and learning disabilities and had received specific and detailed training regarding the administration of the questionnaire. Teachers and parents received separate links for the same questionnaire and were asked to complete it within one week. The study was conducted in the middle of the academic year, between February and April.

## RESULTS

Table 1 presents the descriptive statistics of participants' raw scores for the general (composite) SEL reported by students in different grade levels, their parents, their teachers, with no indication of ceiling or floor effects. Indeed, all variables were screened for skewed measurements in the different groups, showing that all variables were normally distributed (Blanca et al., 2013). The means of SEL skills generally indicated acceptable levels among the three groups, with decreasing trends across grade levels.

**TABLE 1:** Descriptive statistics for SEL skills of students, parents and teachers by grade level.

Grade	Students^a^			Parents^b^			Teahers^c^		
	*M*	SD	Skew	*M*	SD	Skew	*M*	SD	Skew
3^d^	4.12	.34	−.30	4.37	.38	−.93	4.16	.56	−.94
5^e^	3.97	.53	−.83	4.14	.45	−.45	3.61	.80	.02
7^f^	3.80	.52	−.26	4.07	.57	−.49	3.35	.92	−.31
9^g^	3.60	.55	−.24	3.98	.67	−.78	3.26	.78	−.70

Abbreviations: SEL, social–emotional learning; Skew, skewness.

^a^ *n* = 241.

^b^ *n* = 170.

^c^ *n* = 236.

^d^ *n* = 69.

^e^ *n* = 62.

^f^ *n* = 54.

^g^ *n* = 56.

To address our first research question regarding differences in SEL skills perception between students, their parents, teachers across different grade levels, we conducted two‐way ANOVAs with repeated measures. These analyses revealed a significant main effect of group, *F*(2, 168) = 28.5, *p* < .001, *η* ^2^ = .148, grade level, *F*(3, 168) = 3.2, *p* < .01, *η* ^2^ = .056. To identify the source of the group effect, pairwise comparisons using the Bonferroni test were conducted, revealing significant differences (*p* < .01) between the three groups, regardless of grade level. The perceptions of parents (*M* = 4.03) were higher than those of students (*M* = 3.86), and teachers reported lower means (*M* = 3.68) than others, that is, parents > students > teachers. It is noteworthy that despite these differences between groups, there were significant moderate correlations between children and both parents (*r* = .43, *p* < .01) and teachers (*r* = .44, *p* < .01), as well as between parents and teachers (*r* = .54, *p* < .01), regardless of grade level.

**FIGURE 1:**
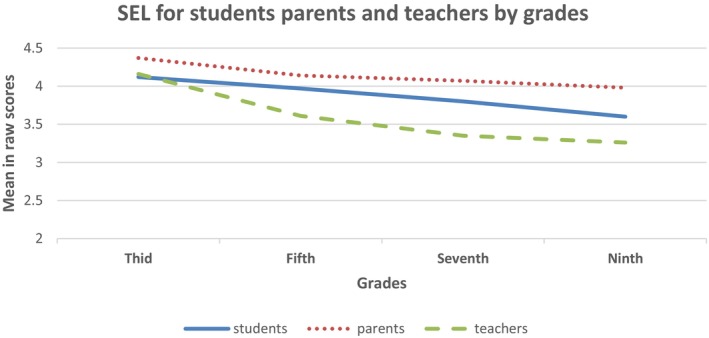
Performance in SEL skills (composite) of the three groups by grade level.

Regarding the effect of grade, pairwise comparisons using the Bonferroni test revealed a decreasing trend with age, regardless of group. However, these differences between grade levels were primarily related to third graders, who reported significantly (*p* < .001) higher scores than all other grades, which did not differ from each other. A significant interaction was found between group and grade level, *F*(2, 168) = 3.23, *p* < .01, *η* ^2^ = .056, which may be related to larger discrepancies between groups with age (see Figure 1). Specifically, the paired sample *t*‐test showed that the differences between students and the other two groups were not significant in the third grade, and students differed only from teachers in the fifth grade. However, parents and teachers differed from each other in these grades. Additionally, all groups differed significantly from each other in the seventh and ninth grades.

To address the second research question and examine the possible influence of discrepancies between groups on the relationship with academic achievements, we conducted a correlation analysis between SEL skills and academic scores separately for the three groups. As presented in Table 2, while moderate significant correlations were found among both students and parents in third and fifth grades, large correlations between SEL skills and academic scores were found among teachers in these elementary grades. With age, the correlation between students' SEL skills and academic scores failed to reach significance starting from seventh grade, and the same occurred for parents' correlations in the ninth grade. Notably, the relationship between teachers' SEL reporting and students' academic scores showed large significant correlations in all grades, with some decrease in the ninth grade. The overall correlations (across grade levels) showed that while students' and parents' SEL reporting were moderately correlated with students' academic scores, large correlations were found between teachers' SEL reporting and students' academic scores.

**TABLE 2:** Summary of intercorrelations between SEL groups and academic scores by grade.

Grade	Academic scores			
	SEL groups	Arabic	Math	Science
Third	Students	.30*	.29*	.25*
	Parents	.34**	.36**	.31*
	Teachers	.71**	.75**	.65**
Fifth	Students	.42**	.39**	.44**
	Parents	.38**	.40**	.39**
	Teachers	.63**	.78**	.71**
Seventh	Students	.18	.25	.15
	Parents	.51**	.47**	.35*
	Teachers	.64**	.72**	.69**
Ninth	Students	.25	.14	.11
	Parents	.01	.05	.08
	Teachers	.44**	.47**	.52**
Overall	Students	.40**	.38**	.35**
	Parents	.40**	.39**	.37**
	Teachers	.66**	.73**	.69**

Abbreviation: SEL, social–emotional learning.

* *p* < .05.

** *p* < .01.

In addition, an in‐depth analysis was conducted to test how the SEL reports predict academic scores among the three groups, regardless of grade level, using structural equation modelling (SEM) analysis. The SEM analysis requires about 25 participants per variable, which does not allow for conducting separate SEM analyses for each grade level. Maximum likelihood estimation procedures were used to analyse the (general) explained variance of the SEL reports as predictors (latent variables) and the academic scores as a dependent variable (latent variable), simultaneously among students, parents, teachers. The dependent latent variables represented the general performance of students' scores, across grades, in Arabic (*M* = 81.2, SD = 17.5), math (*M* = 79.4, SD = 19.4), science (*M* = 80.6, SD = 19.5) as reported in the school administrative records. The three independent latent variables for students, parents, teachers each included five observed variables: self‐awareness, self‐regulation or self‐management, social awareness, relationship skills, responsible decision making. The descriptive statistics of participants' raw scores for the SEL subscales (observed variables) reported by the three groups are presented in Table 3, with no indication of ceiling or floor effects.

**TABLE 3:** Descriptive statistics for SEL subscales of students, parents and teachers.

Grade	Students			Parents			Teachers		
	*M*	SD	Skew	*M*	SD	Skew	*M*	SD	Skew
Self‐awareness	3.99	.73	−.26	4.26	.68	−.84	3.71	1.03	−.54
Self‐regulation	3.28	.38	−.59	3.18	.28	−.30	3.11	.30	.02
Social awareness	4.14	.72	−.62	4.51	.55	−1.22	3.77	.97	−.64
Relationship skills	4.10	.67	−.61	4.47	.54	−1.01	3.94	.75	−.68
Decision making	3.90	.73	−.61	3.97	.69	−.61	3.35	.55	−.39

**FIGURE 2:**
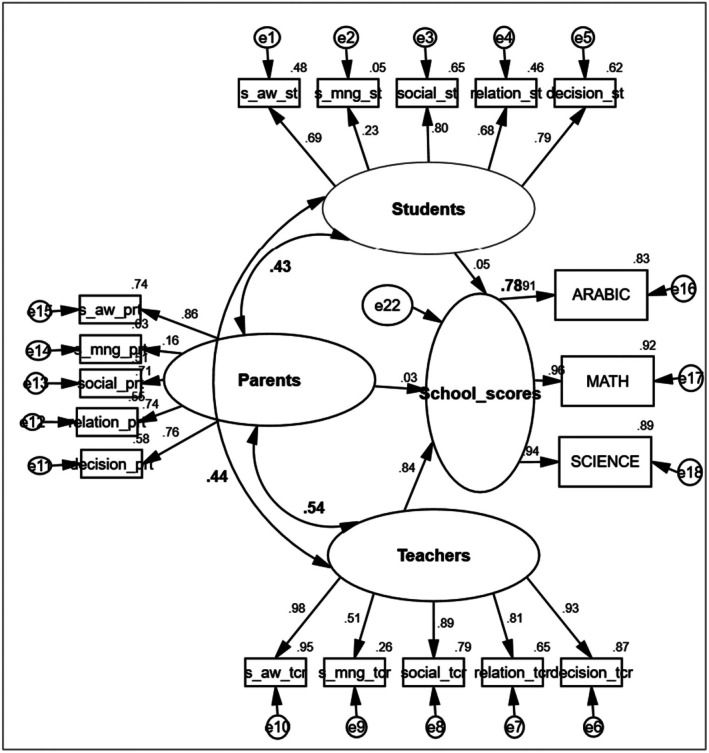
Structural equation modelling (SEM) analysis for predicting students' academic scores by students', parents' and teachers' SEL reporting. Decision, responsible decision‐making; prn, parents; relation, relationship skills; s_aw, self, awareness; s_mng, self‐management; social, social skills; st, students'; tcr, teachers.

The SEM model provided a good fit to the data based on the following fit indices: RMSEA = .06, CFI = .95, TLI = .94. Carrying out simultaneously one model for the three groups provides a more competitive figure between the groups but will provide one general index of explained variance (Figure 2). However, the contribution of each group will be evaluated by the relative standardized coefficients (*β*) of each predictor. The results from the SEM analysis showed that the dependent latent variables of all groups explained 78% of the variance in academic scores (see Figure 1). However, as shown by the standardized coefficients (*β*) of each group, while the relative contribution of teachers' SEL report to academic scores was significant (*β* = .84, *p* < .001), the relative contributions of both students (*β* = .05, *p* > .05) and parents (*β* = .03, *p* > .05) were not significant.

## DISCUSSION

The present study aimed to examine potential discrepancies in perceptions of social and emotional learning (SEL) skills among 241 students from third, fifth, seventh and ninth grades, their parents, their teachers. Analysis of variance (ANOVA) revealed significant discrepancies among the three groups. Furthermore, despite moderate correlations between students' and parents' reports of SEL and academic scores, structural equation modelling (SEM) analyses demonstrated that only teachers' SEL reports significantly predicted students' academic scores.

A key factor influencing perceptions and reporting of SEL within Arabic‐speaking communities is the interplay of cultural values, family dynamics and shared educational experiences (Agbaria, 2022). The observed discrepancies in students' self‐reported SEL skills and their parents' and teachers' ratings may reflect the intricate dynamics of the students–parents–teachers relationship within a marginalized minority population (Avissar, 2025). These dynamics are embedded within a socio‐political context characterized by historical marginalization, persistent structural inequalities, limited cultural representation in mainstream education (Avissar, 2025). In particular, among Arab minorities in Israel, close familial relationships, strong group identity, unique socialization practices shape how students, parents, teachers perceive and evaluate social and emotional competencies (Im, 2023; Albritton et al., 2024). For instance, parental authority, expectations for respect, variation in parenting styles (Agbaria, 2022) contribute to divergent assessments, particularly when parents prioritize behaviours aligning with cultural norms rather than those emphasized in Western‐oriented SEL frameworks (Suárez‐Orozco et al., 2018). Teachers, by contrast, operate within an educational system predominantly informed by majority cultural standards that emphasize autonomy, self‐expression, individual self‐regulation. The perspectives of teachers are further influenced by professional training and by social and political challenges faced by Arab communities within Israeli schools (Paul‐Binyamin, 2024; Ramon, 2021).

Our findings regarding the discrepancies between students' self‐reported SEL skills and their parents' and teachers' ratings align with several previous studies (Caqueo‐Urízar et al., 2022; De Los Reyes et al., 2015; Feng et al., 2022; Renk & Phares, 2004). This consistency is further reflected in the moderate correlations found in this study between students' self‐reported SEL skills and their parents' and teachers' ratings. The discrepancies reported here, particularly regarding students, do not appear to stem from social desirability bias, as these discrepancies are not in favour of the student group. Indeed, the scores of the SEL (composite) skills reported by students, regardless of grade level, were lower than their parents' ratings. When examining these discrepancies within grade levels, students' self‐reports were similar to those of their parents' ratings in the third and fifth grades and even lower than their parents' ratings in the seventh and ninth grades. This pattern may be attributed to the fact that younger students in elementary school spend more time with family at home, providing parents with more opportunities to interact with them and gain insight into their social–emotional functioning. In contrast, adolescents spend more time away from home and share less information with parents about their emotional experiences and social interactions.

Discrepancies between parents and teachers have also been reported in previous research (De Los Reyes et al., 2015; Martinsone et al., 2022), suggesting that student behaviours may vary across settings and contexts (Hartley et al., 2011; Rosenthal et al., 2025). Such discrepancies may be further explained by teachers' professional training and experience in child development, which provides a more objective and comprehensive basis for evaluation than parental perspectives (Dicke et al., 2015; Masry‐Herzallah, 2025). In addition, teachers are uniquely positioned to observe students in peer‐social environments (Stone et al., 2010), enhancing their predictive accuracy regarding cognitive and non‐cognitive outcomes (Feng et al., 2022; Verhulst et al., 1997). Conversely, parents motivated by affective concerns and aspirational hopes for their children, may offer more favourable ratings relative to teachers, whose observations tend to be more dispassionate (De Los Reyes et al., 2015). Additionally, parental ratings may be influenced by high expectations, a feature associated with authoritative parenting (Ren & Pope Edwards, 2015), which is characteristic of the Arab community in Israel. Notably, a large sample of 420 Arab‐speaking mothers revealed a significant association between authoritative parenting style and elevated child SEL skills (Agbaria & Mahamid, 2023). It should also be noted that the degree of agreement among raters is higher among younger children than older students (De Los Reyes et al., 2015).

Regarding age differences, our results revealed a decreasing trend in self‐reported SEL (composite) skills with age, regardless of group, which was primarily reflected between the earliest grade and all others. This declining trend in SEL skills aligns with previous studies (Berg et al., 2021; Pan et al., 2023). This pattern may be related to changes in relationships and closeness between students and their parents and teachers as children age, becoming less solid in adolescence, which is recognized as one of the most psychologically challenging periods of development (Orben et al., 2022). Indeed, SEL skills do not develop monotonically with age but have been shown to follow a U‐shaped trajectory, decreasing from 12 to 16 years of age and increasing thereafter (Feraco & Meneghetti, 2023).

The impact of discrepancies among groups reported in our study was reflected in the correlation analysis between SEL reporting of the three groups and students' academic scores, with larger correlations observed in the teacher group. Additionally, the SEM model of prediction of SEL skills to students' academic scores, regardless of grade level, demonstrated strong predictions of academic scores among teachers, while students' and parents' predictions were not significant. This pattern was also reflected in the decreasing trend in the correlations between students' and parents' SEL reporting and students' academic scores. Our results support previous findings that revealed a clear advantage of teachers' SEL reporting in predicting students' academic scores (Feng et al., 2022; Kuhfeld et al., 2023).

This advantage underscores the unique role that teachers play in evaluating SEL skills in educational settings. The powerful prediction of teachers may be attributed to their professional training, which provides them with a comprehensive view of how SEL skills should be developed and manifested in the classroom, along with a deep understanding of how these skills influence students' academic performance (Jennings & Greenberg, 2009). Furthermore, the context of evaluation may also contribute to this advantage, as teachers evaluate students' SEL skills in the same school setting in which academic scores are assessed.

In conclusion, our comparison of discrepancies between Arabic‐speaking students, their parents, their teachers regarding students' self‐reported SEL skills revealed significant differences in perceptions among the three groups. Teachers reported the lowest scores, while parents reported the highest. Additionally, a declining trend in SEL skills with age was observed, regardless of group. These discrepancies were also reflected in the relationships between students' SEL skills and their academic scores, with teachers' SEL skill evaluations showing larger correlations with academic scores compared to the other two groups. Notably, only teachers' SEL ratings significantly predicted students' academic scores.

This study has several theoretical and pedagogical implications. The significant correlations between SEL skills and academic scores suggest that SEL should be more thoroughly integrated into curricula, subject lessons, school activities, particularly among Arabic‐speaking students, which may positively influence both their cognitive and non‐cognitive performance. The clear discrepancies among students, parents, teachers highlight the importance of developing a multi‐informant method for SEL evaluation, supporting the ecological systems theory's view emphasizing the need for multiple contexts to better understand students' development. Furthermore, evaluative contexts must be acknowledged, as behaviour is shaped by environmental factors and culturally adapted SEL programmes have been shown to enhance interpersonal skills, prosocial behaviours and positive attitudes, addressing the inherent challenges of cross‐informant assessment (Im, 2023; Albritton et al., 2024). More specifically, considering cultural and social nuances of the Arabic community—including language, religion, minority status, systemic inequalities—SEL assessment and intervention programmes must be tailored to the unique context of Arab communities in Israel (Agbaria, 2024). Ensuring SEL curricula reflect local cultural, linguistic and historical identities, for instance by incorporating community traditions, stories, native languages, is essential. Schools should actively engage parents and community members as authentic partners in the ongoing development and implementation of SEL initiatives, ensuring local values and voices inform programme design and objectives.

The critical role of teachers was highlighted by their predictive power, necessitating more comprehensive professional training programmes that enhance teachers' capability to observe and evaluate, as well as foster SEL skills in classrooms alongside academic performance. Furthermore, increased communication among students, parents, teachers regarding SEL development, importance, expectations may contribute to greater alignment of perceptions across different contexts. This is particularly crucial for advanced grade levels, where the relationships between students' and parents' SEL reporting and students' academic scores appear to diminish.

The main limitation of this study is related to the sample size, particularly when considering different grades and developmental effects. The limited number of participants did not allow for conducting SEM analyses separately for each grade level, as each variable in this analysis requires approximately 25 participants. Future developmental studies with larger sample sizes may provide more accurate data for developing standardized SEL questionnaires for different age groups of Arabic‐speaking populations. Additionally, the restriction of sampling to two villages limits the generalizability of findings to the broader Arabic‐speaking population. Finally, the exclusive reliance on self‐report questionnaires presents inherent challenges in measurement. Subsequent studies are advised to utilize expanded and diversified samples, complemented by mixed‐method approaches to enhance representativeness and validity.

## AUTHOR CONTRIBUTIONS

**Ibrahim Asadi:** Conceptualization; investigation; writing – original draft; methodology; validation; visualization; writing – review and editing; software; formal analysis; project administration; data curation; supervision; resources.

## CONFLICT OF INTEREST STATEMENT

The authors declare that they have no conflict of interest and that no funding was received.

## INFORMED CONSENT

Informed consent was obtained from all individual participants included in the study.

## Data Availability

Information regarding the data and conditions for access are available from the corresponding author.
